# Novel Isoforms of the Transport Regulator Klar

**DOI:** 10.1371/journal.pone.0055070

**Published:** 2013-02-14

**Authors:** Dae-Hwan Kim, Sean L. Cotton, Dipak Manna, Michael Welte

**Affiliations:** 1 Department of Biology, Brandeis University, Waltham, Massachusetts, United States of America; 2 Department of Biology, University of Rochester, Rochester, New York, United States of America; Instituto Gulbenkian de Ciência, Portugal

## Abstract

Klar is a regulator of microtubule-motor dependent transport processes in *Drosophila*, including nuclear migration, vesicle motility, and lipid-droplet transport. The single *klar* locus gives rise to multiple isoforms that presumably have unique functions. Up to now, three Klar isoforms (α, β, γ) were known. Here we describe two novel isoforms, δ and ε, whose expression depends on a previously uncharacterized promoter. Klar δ and/or ε are widely expressed during development, including in the embryonic and larval nervous system as well as in ovaries. When we specifically ablate Klar δ and ε expression genetically, no gross organismal phenotypes are apparent. However, ectopic expression of these isoforms causes nuclear mispositioning in developing photoreceptors and in oocytes, demonstrating their biological activity. Our analysis identifies novel forms of the Klar protein and provides new tools for functionally dissecting the complex *klar* locus.

## Introduction

Klarsicht (Klar) is an important regulator of intracellular trafficking in *Drosophila* and has been proposed to either anchor microtubule motors like cytoplasmic dynein to cargoes to be moved [1] or to coordinate the activity of opposing motors kinesin-1 and dynein attached to a single cargo [2]. Although Klar's primary sequence is not obviously conserved beyond arthropods, it has been suggested to be functionally analogous to *C. elegans* Unc-83 and mammalian Nesprin-4 [3]: At least some isoforms of each of these proteins carry nuclear-envelope targeting KASH domains and play roles in nuclear-positioning events in specific cell types. All three proteins have been shown or interfered to physically interact with either kinesin-1 or dynein or both [3]–[5] and have been proposed to act as motor anchors or motor coordinators [2], [6]–[8].

During *Drosophila* development, Klar mediates a number of specific morphogenetic processes. Klar is required for the basal-to-apical migration of nuclei in larval photoreceptors [2], [6] and regulates lipid-droplet motion in early embryos [2], [9]. Klar also promotes expansion of the apical membrane in embryonic salivary glands [10], helps to establish the even spacing of myonuclei in embryos and larvae [11], and contributes to the localization of RNP particles to the posterior pole of oocytes (Yu et al., unpublished results). In addition, indirect evidence suggests roles of Klar in wing development [12], in neuroendocrine cell remodeling [13], branch migration in trachea [14], and starvation stress resistance [15].

How does Klar act specifically in so many distinct transport processes? This adaptability is, at least in part, due to isoform variation: The single *klar* locus gives rise to at least three distinct isoforms with different functions: α, β and γ [6], [9]. Nuclear migration in eye discs depends on endogenously expressed Klar α [6], [16], [17], but does not require Klar β [9], [18]. Klar α is also implicated in membrane trafficking in salivary glands [10]. Lipid-droplet motion in embryos requires Klar β, but not Klar α or Klar γ [9], [18]. So far, no function has been attributed to Klar γ. Isoform variation has also been described for Unc-83 and Nesprin-4, suggesting that these regulators use a similar strategy to generate functional diversity [19], [20].

**Figure 1:**
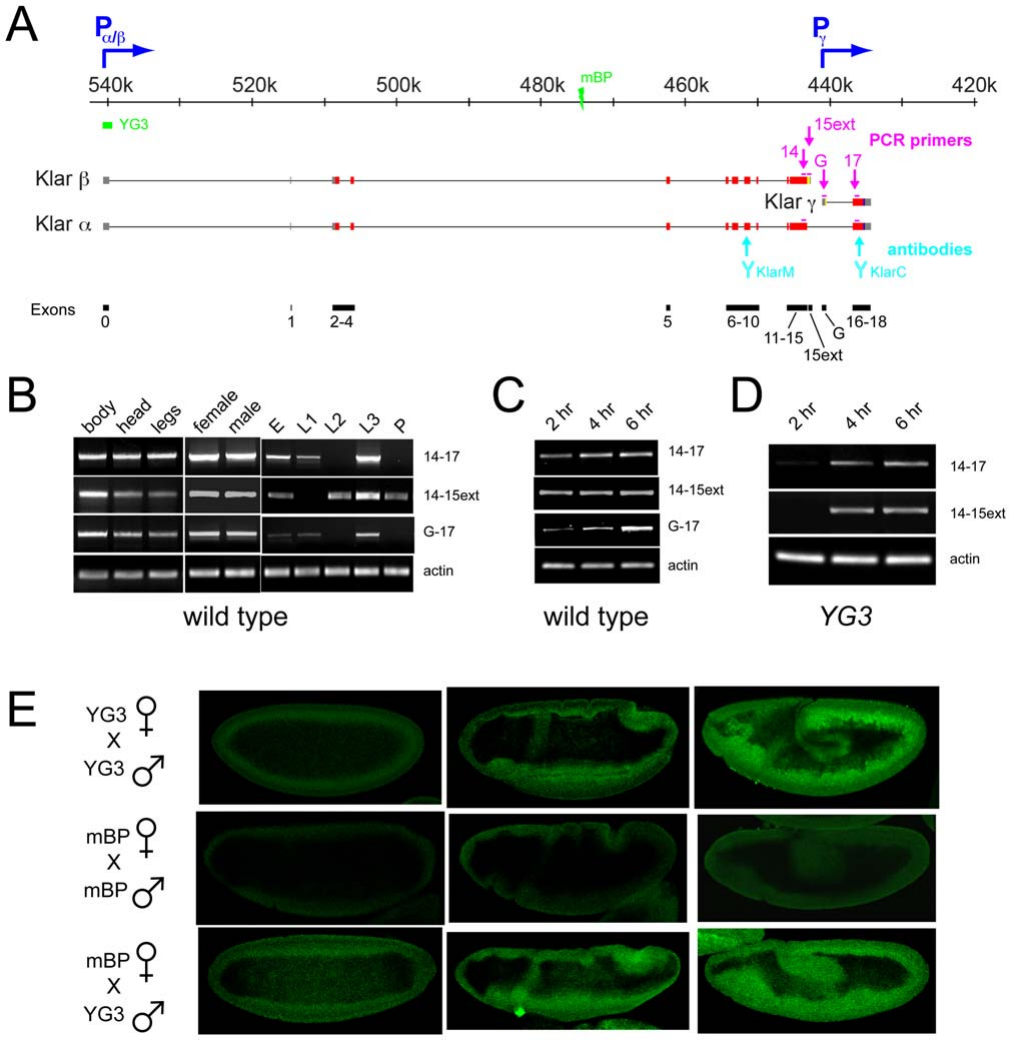
Early embryos express a novel form of Klar. (A) Schematic representation of the *klar* locus. Promoters are indicated by blue arrows, non-coding exons by gray bars, and coding exons by red bars. Coding regions unique to a single Klar isoform (15ext and G) are shown in yellow. Blue  =  KASH domain. The location of antibody epitopes (cyan) and PCR primers (purple) is shown, as well as the lesions in the two alleles *klar^YG3^* (promoter deletion) and *klar^mBP^* (chromosomal break). Numbers indicate nucleotide position on chromosome 3. Exon numbering is shown at the bottom (black). (B) RT-PCR analysis for Klar in adult body parts (left), entire adult males and females (middle), and throughout development (right): 0–4 hr old embryos (E), larval instars (L1, L2, L3), and pupae (P). No signal was detected in animals lacking the *klar* locus entirely (data not shown). Actin primers were used to confirm equal loading. (C) Klar expression in early embryos, 2, 4 and 6 hrs old, analyzed as for (B). (D) *klar^YG3^* embryos express *klar* mRNAs. RT-PCR analysis of equal amounts of RNA extracted from 2 hr, 4 hr, and 6 hr old embryos, as for (B). (E) Klar protein accumulates in *klar^YG3^* embryos due to zygotic expression of Klar. Embryos from various crosses were fixed and stained with Klar-M antibody. In *klar^YG3^* embryos (top), no Klar-M signal is detectable in cycle 14 (left), but becomes apparent in early (middle) and late (right) germ-band extension stages. Absence of such signal in *klar^mBP^* embryos (middle) demonstrates that the signal is Klar specific. When *klar^mBP^* mutant mothers are crossed to *klar^YG3^* mutant fathers, embryos accumulate Klar-M signal during germ-band extension (bottom).

Isoform variation in Klar is achieved by the use of multiple promoters and alternative splicing (Fig. 1A). The message for the α isoform spans the entire ∼110 kb *klar* locus and contains nineteen exons (labeled exon 0 through 18). Genetic analysis [9], [18] and subsequent cloning (Kim et al., unpublished results) of the Klar β message demonstrated that it shares exons 0 through 15 with the Klar α message, and contains a unique 590 nucleotide extension at its 3′ end (exon15ext), generated via alternative splicing. Klar α and β messages are apparently transcribed from a common promoter (P_α/β_) since the *klar^YG3^* allele, a small deletion centered around exon 0 (Fig. 1A), abolishes expression of both isoforms (Yu et al., unpublished results; see also below). The promoter of the γ isoform (P_γ_) is ∼1.5 kb downstream of exon15ext. The γ cDNA contains a unique 5′ exon G and shares exons 16, 17 and 18 with klar α [9].

Both the *klar* mutant phenotypes and available immunostaining data [9], [21] suggest that Klar is widely expressed, though in only a few instances has it been identified which Klar isoforms are responsible. We therefore undertook a comprehensive description of the developmental expression of Klar α, β and γ. In the course of these studies, we uncovered previously uncharacterized Klar isoforms (δ and ε) and found that in some cases multiple isoforms are expressed simultaneously. Combinatorial deployment of distinct Klar isoforms may allow cells to further adapt Klar functions to particular tasks. To be able to study the function of these two isoforms, we generated a deletion of the promoter responsible for δ and ε expression and identified a transposable element insertion near the promoter that allows ectopic δ/ε expression. While loss of δ/ε had no discernable effects on development or nuclear positioning in eye discs, ectopic expression severely disrupted positioning of nuclei in photoreceptors and oocytes. These data reveal that these isoforms can have potent biological activity. Our analysis provides important new tools for dissecting Klar function.

## Results

### Klar α, β and γ are expressed throughout *Drosophila* development

Previous Western and immunolocalization studies have detected Klar expression in specific embryonic, larval, and adult tissues [9], [18], [21], [22], but no comprehensive developmental time course has yet been reported. In addition, detection of the protein by immunostaining makes it challenging to identify which isoform is expressed, in part because available antibodies each recognize two of the isoforms [9] (see also Fig. 1A).

We therefore designed primer sets to distinguish the mRNAs for the three isoforms by RT-PCR (purple in Fig. 1A). We used primer pairs in exons 14 and 17 to detect Klar α (predicted product: 550 bp), in exons 14 and 15ext to detect Klar β (predicted product: 450 bp), and in exons G and 17 to detect Klar γ (predicted product 450 bp).

For all three probes, we detected amplification products of the correct size across many developmental stages (Fig. 1B). For adults, we detected robust signal in both males and females as well as in fractions enriched for bodies, heads, and legs. Signal was also detected throughout development, from embryos through larvae and pupae, though the signal among the three probes was divergent.

During embryonic development, the Klar isoforms displayed a dynamic expression pattern (Fig. 1C). We collected embryos for 2 hours and aged the collection for various times. Most of the RNAs present in 0–2 hours embryos are generated during oogenesis, and previous *in-situ* and Western analysis had indeed revealed that Klar β message and protein are maternally provided [9]. Changes in older embryos would reflect degradation of such maternal pools and/or new transcription. While the Klar β probe yielded similar signal at all three time points, signal for Klar α and especially Klar γ increased dramatically over time, implying new transcription for these isoforms during the first 6 hours of embryogenesis (Fig. 1C). Published microarray studies with probes not designed to distinguish isoforms confirm that Klar expression is very dynamic during development, especially during pupal stages [23]. This pattern implies that Klar is required for specific, developmentally controlled processes, and thus likely plays roles beyond its characterized functions.

### Zygotic expression of Klar is in part due to a previously uncharacterized transcription start site

The changes in Klar α and Klar γ signal (Fig. 1C) suggest that Klar transcription is highly regulated during early embryogenesis. A careful analysis of the temporal and spatial pattern of this expression might reveal new processes in which Klar plays a role. However, teasing out the contribution of new mRNA and protein expression in early embryos is challenging because there is substantial maternal deposit of Klar message and protein. This maternal pool might mask the contribution of new expression.

By analyzing females lacking Klar expression, it is possible to remove this maternal pool. Zygotes derived from such females and wild-type males should still be able to express Klar zygotically, and thus they should allow pinpointing the spatial and temporal contribution of zygotically generated Klar. In particular, we employed females homozygous for the *klar^YG3^* allele, a deletion of the P_α/ β_ promoter (Fig. 1A). It indeed abolishes Klar α and β protein expression in ovaries; Klar γ expression remains intact [9]. Early embryos derived from *klar^YG3^* mothers and fathers also lack detectable Klar α or β messages (as detected by in situ hybridization [9] and by RT-PCR (Fig. 1D, 2 hr sample)) or Klar protein [9], [18].

**Figure 2:**
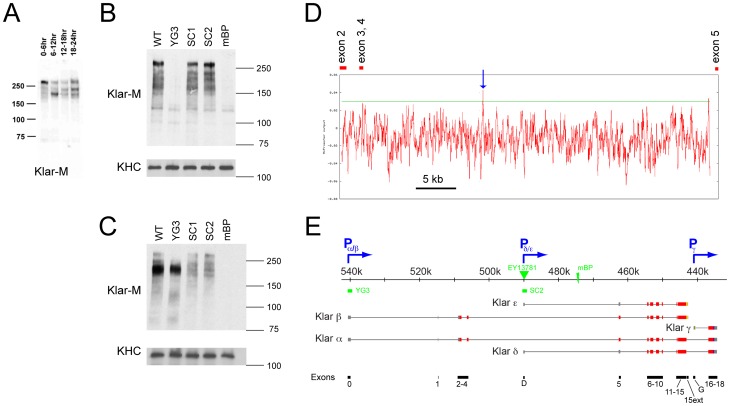
Identification of the P_δ/ε_ promoter. (A) Klar protein expression pattern varies with embryo age. Embryos were collected for six hours, aged for various times, and heat-fixed. Proteins from equal numbers of embryos were analyzed by Western Blotting with Klar-M. In 0–6 hr embryos, a major band corresponding to the β isoform is present above the 250 kD molecular weight marker. In 6–12 hr embryos, a new major band appears at ∼210 kDa. (B and C) Klar-M Western blots of embryos of various genotypes. After heat-fixation, embryos in cycle 14 (B) or germ-band extension (C) were recovered and analyzed by Western Blotting as in (A). Detection of KHC (kinesin heavy chain) serves as loading control. (D) Read-out of the McPromoter program [24], [25] for the *klar* genomic region from exon 2 to exon 5. The prediction score representing likelihood for a promoter (red line) rises above the threshold (green) only at a single location (blue arrow). (E) Updated cartoon of the *klar* locus. Color scheme and symbols are as in Fig. 1A. The locus encodes five different isoforms: expression of Klar α and β is driven by promoter P_α/β_, Klar δ and ε by P_δ/ε_, and Klar γ by P_γ_. Insertion of the P element *EY13781* (green triangle) and deletion of the predicted P_δ/ε_ promoter (*klar^SC2^*) abolish expression of Klar δ and ε. Exon numbering is shown at the bottom (black).

Unexpectedly, when we examined later stages of the same genotype, we detected RT-PCR signal with both our Klar α and β probes (Fig. 1D). This result suggests that at least some of the zygotic expression of Klar detected in the wild type is due to an as-yet uncharacterized *klar* promoter. It appears to drive expression of messages with two different 3′ ends.

To determine if this expression leads to the production of Klar proteins, we examined *klar^YG3^* embryos by immunostaining with Klar-M, a Klar-specific antibody that recognizes an epitope in exon 9 [9] (see also Fig. 1A). In early embryos until cellularization, we detected no signal above background (Fig. 1E), as previously described [9]. However, during gastrulation and germ-band extension, the signal increased dramatically.

If this antibody reactivity indeed represents a novel form of Klar not dependent on the P_α/β_ promoter responsible for α/β expression, then it is presumably driven by a more proximal promoter, located somewhere between exon 0 (deleted in *klar^YG3^*) and exon 9 (the epitope recognized by antibody Klar-M). We therefore examined a distinct *klar* (*klar^mBP^*) allele that is due to a translocation in which the chromosome is broken between exons 4 and 5 (Fig. 1A) [6]. In embryos of this genotype, we detected no signal above background even during germ-band extension (Fig. 1E). This result demonstrates that the signal observed in *klar^YG3^* is indeed Klar specific, and suggests that expression is due to a promoter that is upstream of the *klar^mBP^* chromosomal break point.

The *klar^mBP^* allele allowed us to perform a variant of the experiment initially imagined since this allele on its own abolishes both maternal and zygotic Klar expression. We therefore crossed *klar^mBP^* mothers to *klar^YG3^* fathers and examined their embryos. We observed similar Klar expression pattern as when both mothers and fathers were homozygous for *klar^YG3^*, demonstrating that this allele zygotically expressed a form of Klar (Fig. 1E).

### Zygotic *klar* expression results in new Klar protein isoform(s)

The translation start for Klar α and β is located in exon 2, more than 30 kb downstream of the transcription start site for the α and β messages and the region deleted in *klar^YG3^* [6], [9] (Fig. 1A). It is therefore conceivable that a promoter upstream of exon 2 drives the Klar expression we observed in *klar^YG3^*, resulting in production of the same protein isoforms previously described. Alternatively, a more proximal promoter and the use of different translation starts would result in Klar proteins lacking N-terminal regions present in Klar α and β.

By Western analysis of early wild-type embryos, Klar is detected as a series of proteins of varying molecular weight, with the major band of apparent molecular weight >250 kDa representing Klar β [9]. The identity of the minor bands is unknown, but they all originate from transcription starting at the canonical α/β promoter, since they are absent in *klar^YG3^* embryos; likely, they are derived from full-length Klar β by degradation or proteolytic processing.

To address the identity of the zygotically expressed Klar, we first compared protein samples from wild-type embryos of various ages by Western analysis, using Klar-M (Fig. 2A). The 0–6 hr sample is predominated by the major Klar β band, as seen before [9]. However, in samples of older embryos multiple additional bands of various sizes were detected, including a prominent band of ∼215 kDa in 6–12 hr embryos (Fig. 2A). This band was absent in *klar^mBP^* embryos but present in *klar^YG3^* embryos, and thus represents a form of Klar not expressed from the canonical α/β promoter (Fig. 2C). Its apparent molecular weight is significantly shorter than that of the Klar β present in early embryos. This band also does not represent Klar α (whose molecular weight is even larger than that of Klar β) or Klar γ (∼75 kDa, and is not detected by Klar-M antibody). Thus, it apparently represents a novel Klar isoform distinct from α, β and γ. The Klar-C antibody also recognizes a band of similar size in 6–12 hr embryos, a band absent in *klar^mBP^* (data not shown); thus, this novel isoform (or at least one such isoform) apparently encompasses exon 9 (Klar-M epitope) and exon 18 (Klar-C epitope) (Fig. 1A).

### Mapping a promoter for the new Klar isoforms

Attempts to isolate cDNAs for the novel Klar isoform proved unsuccessful; however, that is not surprising given that we also were not successful at amplifying early exons of the Klar β message even in the wild type. Presumably, the low expression level and great length of these *klar* messages make recovery of 5′ exons particularly difficult.

However, our analysis suggested an alternative strategy for identifying the transcription start site of this unknown isoform: First, since the novel isoform shares exons 9 and 18 with Klar α, but has a much shorter apparent molecular weight by Western analysis, it presumably lacks N-terminal sequences relative to Klar α, and thus its translation likely starts downstream of exon 2. Second, this isoform is absent in *klar^mBP^* animals, and thus transcription for it likely starts upstream of the breakpoint of *klar^mBP^* (Fig. 1A).

We therefore examined the region between exon 2 and exon 5 for potential promoters (Fig. 2D), using the McPromoter prediction program [24], [25]. At the highest sensitivity setting, only one potential transcription start site, located ∼15 kb downstream of exon 4, was predicted above threshold (marked as promoter P_δ/ε_ in Fig. 2E). Two independent P-element insertions (*EY13781*, *P{GSV6}GS11451*) have been reported within 1 kb of this potential transcription start site, consistent with the fact that P elements tend to insert into promoter regions [26]. The genomic region downstream of the predicted promoter (called exon D in the following) is indeed transcribed: in various RNA seq datasets, this region shows robust signal above background [27]. In addition, the exon D-exon 5 junction has been found by RNA-seq analysis (FlyBase ID FBsf0000106532), and we recovered a piece of cDNA containing exon D, exon 5, and exon 6 sequences from an embryonic cDNA library (data not shown).

Conceptual translation of exon D revealed numerous stop codons in all three reading frames, suggesting that this exon is part of the 5′ UTR. In addition, neither exons 5 nor 6 had start codons in the reading frame corresponding to Klar α and β. This analysis predicts that protein isoforms expressed from the hypothetical P_δ/ε_ promoter start with amino acid 630 (in exon 7) of the canonical Klar α sequence and, compared to Klar α and β, lack N-terminal sequences of ∼ 68 kDa, consistent with the difference in apparently mobility we detect on Westerns (Fig. 2A, B, C). We designate this promoter P_δ/ε_ because it can apparently drive expression of two different messages, provisionally called δ and ε, with alternative 3′ ends (Fig. 2E).

To abolish expression from the P_δ/ε_ promoter, we employed imprecise-excision of the *EY13781* P-element and recovered allele *klar^SC2^* that removes 2040 bp of genomic sequence, including the predicted promoter. By Western analysis, both *klar^SC2^* and the original P insertion (in the following referred to as *klar^SC1^*) greatly reduce – and possibly entirely abrogate – expression of the ∼215 kDa Klar form in embryos in germ-band extension (Fig. 2C), while leaving expression of Klar β intact (Fig. 2B).

### Klar expression from the P_δ/ε_ promoter is widespread during *Drosophila* development

**Figure 3:**
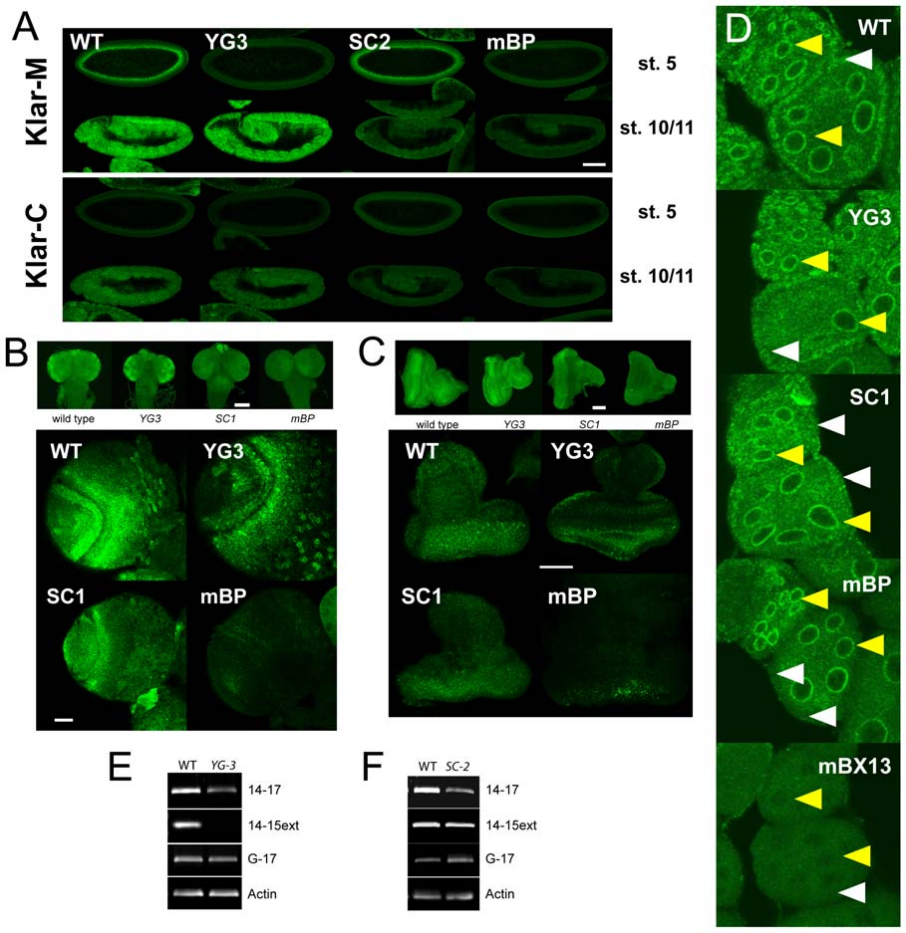
The P _δ/ε_ **promoter contributes to Klar expression in various developmental stages.** (A) Klar-M (top) and Klar-C (bottom) staining of stage 5 and stage 10/11 embryos. (B, C) Klar-M staining in, larval brains (B), and eye imaginal discs (C). The allele *klar^mBP^* serves as negative control. (D) Klar-C staining in early egg chambers. Wild-type and *klar^YG3^* animals show abundant Klar signal in nurse cells (especially perinuclearly, yellow arrowheads) and in follicle cells (white arrowheads). Follicle cell signal is reduced or entirely absent in *klar^SC2^* or *klar^mBP^* animals, respectively. The allele *klar^mBX13^* serves as negative control. (E, F) RT-PCR signal for Klar isoforms is reduced in both *klar^YG3^* and *klar^SC2^*. Signal for the γ isoform and for actin are unchanged. Scale bars: 100 µm (A), 80 µm (B, top), 25 µm (B, bottom), 40 µm (C, top), 50 µm (C, bottom).

In the wild type, Klar protein expression (as detected by Klar-M or Klar-C staining) displays complicated temporal and spatial patterns throughout development [9]. In stage 5 embryos, Klar signal accumulates around the central yolk, while during germ-band extension, Klar is widely distributed, and is particularly strong in a repeating pattern suggestive of the developing nervous system (Fig. 3A). In third instar larvae, Klar is detected in the brain (enriched in brain lobes, Fig. 3B) and in eye imaginal discs (enriched in regions posterior to the morphogenetic furrow, Fig. 3C). This signal is Klar-specific, as it is absent in *klar^mBP^* animals. In ovaries, Klar is abundant in follicle cells, nurse cells, and oocytes [9]. For example, in early egg chambers (Fig. 3D), Klar-C signal is present strongly around the nuclei of follicle cells (white arrowheads) and nurse cells (yellow arrowheads). In this case, *klar^mBX13^* serves as negative control, as *klar^mBP^* animals retain expression of the γ isoform [9].

In all these tissues, Klar-M and Klar-C show partially overlapping and partially distinct patterns (compare, for example, Fig. 3A, top and bottom panels), reflecting the expression of different mixes of isoforms. For example, in stage 5 embryos, Klar-M signal is strong, but Klar-C signal is close to background, since here Klar expression is dominated by Klar β [9], [18]. And in the nurse cells of early egg chambers, Klar-M signal (not shown) is much less distinct than Klar-C signal [9] (see also Fig. 3D); this is in part because of the expression of Klar γ, an isoform recognized by Klar-C, but not Klar-M (Fig. 1A). This contribution is, for example, apparent from the comparison of *klar^mBP^* and *klar^mBX13^* animals (Fig. 3D): the former retain expression of the γ isoform, the latter do not.

In *klar^YG3^* animals, the wild-type Klar expression pattern is partially abolished. In stage 5 embryos, Klar staining is almost completely abolished, while in stage 10/11 embryos it looks grossly normal (Fig. 3A). In third instar larvae, Klar-M signal is detected throughout eye discs, with less obvious posterior enrichment than in the wild type (Fig. 3C), and prominently in the brain (Fig. 3B). And in early egg chambers, Klar-C still detected prominent signal in follicle cells (Fig. 3D).

Animals homozygous for either *klar^SC1^* or *klar^SC2^* displayed a distinct loss of Klar expression. In embryos during stage 10/11, overall Klar signal was much reduced, (Fig. 3A). In larval eye discs, Klar-M staining was strong anteriorly, but reduced in the rest of the disc (Fig. 3C), and in the larval brain, large rings suggestive of perinuclear signal were absent (Fig. 3B). And in early egg chambers, perinuclear signal in follicle cells was reduced, while perinuclear signal in nurse cells was robust (Fig. 3D). A comparison to the *klar^YG3^* pattern suggests that Klar signal in the wild type is a combination of expression from the P_α/β_ and the P_δ/ε_ promoters.

RT-PCR analysis on adult samples supports this conclusion (Fig. 3E, F). In the wild type, we detected robust signal with all of our three probes (exon 14/17, exon 14/15ext, exon G/17). Klar γ expression (as detected by the G/17 probe) is apparently unaffected in *klar^YG3^* and *klar^SC2^* animals, as expected, since the lesions in these alleles are far from P_γ_. 14/17 signal was reduced in both *klar^YG3^* and *klar^SC2^* animals, suggesting that in the wild type it detects a mixture of Klar α and Klar δ. 14/15ext signal was unaltered in *klar^SC2^* and abolished in *klar^YG3^* animals; apparently, Klar ε makes a negligible contribution to overall Klar levels in adults.

### Phenotypes associated with disruption of the new Klar isoforms

**Figure 4:**
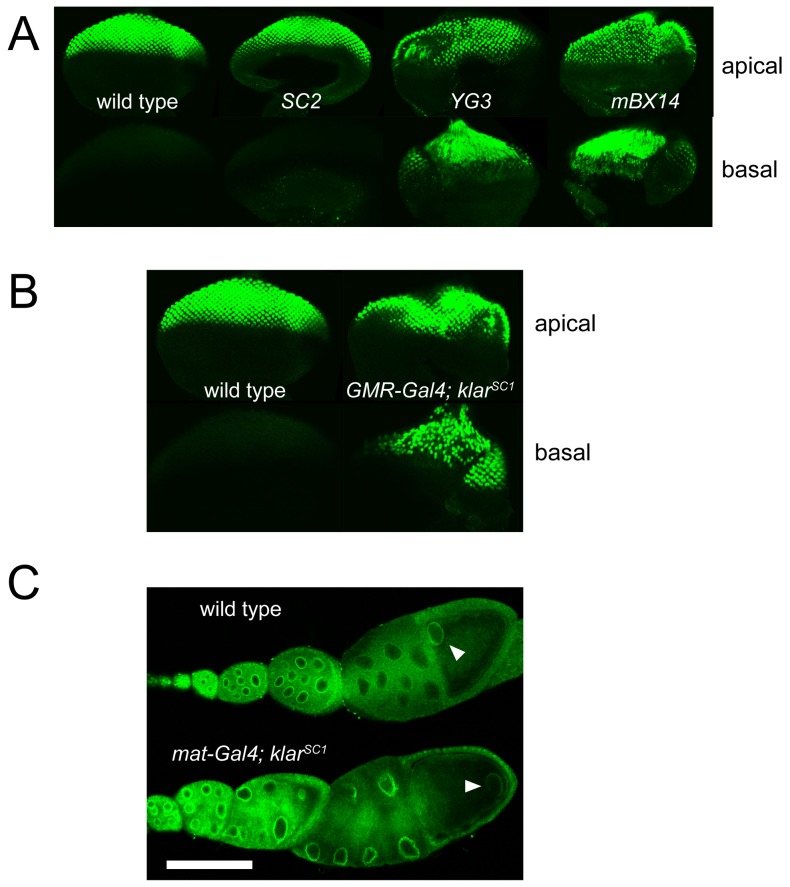
Ectopic expression of Klar δ/ε, but not their absence, causes nuclear mispositioning in eye imaginal discs and oocytes. (A) Eye discs from third-instar larvae were fixed, stained with anti-Elav to reveal photoreceptor nuclei, and examined by confocal microscopy. In wild type and *klar^SC2^*, the photoreceptor nuclei are present only in apical positions. In *klar^YG3^* and *klar^mBX14^*, many nuclei are found basally. (B) Ectopic expression from the P_δ/ε_ promoter results in basal mispositioning of photoreceptor nuclei. Eye discs from wild-type (left) and *GMR-Gal4 klar^SC1^* (right) third-instar larvae, analyzed as in (A). (C) Mid-stage egg chambers from wild-type females (left) and females, in which Klar expression is driven ectopically from the P_δ/ε_ promoter (right). Egg chambers were heat fixed, stained with Klar-C, and analyzed by confocal microscopy. Scale bar  = 150 µm. Arrowheads identify oocyte nuclei.

Animals homozygous for the *klar^SC2^* allele are viable and fertile, and we have not noticed any obvious developmental defects. Because Klar α is crucial for nuclear migration in larval photoreceptors [6], [9] and the new Klar isoforms are co-expressed with Klar α in eye discs (Fig. 3C), we examined the positioning of photoreceptor nuclei in *klar^SC2^* eye discs (Fig. 4A). Eye discs were fixed, stained for Elav to reveal photoreceptor nuclei, and apical and basal sections of the tissues were imaged. In the wild type, all nuclei are apical, arranged in a regular pattern [28]. Disruption of Klar α with the *klar^YG3^* allele results in a disrupted apical pattern, and accumulation of nuclei in basal sections, indicating disrupted nuclear migration (Fig. 4A). Similar disruption is seen with other *klar* alleles [7], [9], *e.g.* a premature stop codon in exon 8 (*klar^mBX14^*) (Fig. 4A). In contrast, *klar^SC2^* eye discs were indistinguishable from the wild type, displaying a regular apical pattern and absence of nuclei in basal sections (Fig. 4A). Thus, the new isoforms expressed from the P_δ/ε_ promoter are apparently not essential for proper nuclear positioning in eye discs.

These novel Klar isoforms may act in a redundant pathway, or their lack may have subtle effects not detectable as gross deficiencies. In such cases, it is often informative to ectopically express the molecule of unknown function to uncover potential biological activities. We took advantage of the fact that the transposable element insertion of *klar^SC1^* carries a UAS element (a binding site for the transcription factor Gal4) as well as a basal promoter and thus should allow expression of neighboring sequences in a Gal4-dependent manner. Indeed, when combining *klar^SC1^* with tissue-specific Gal4 drivers, we observed increased Klar-M and Klar-C signal by immunostaining in the relevant tissues (Fig. 4C, and data not shown).

In particular, we employed the Gal4 drivers elav-Gal4 and GMR-Gal4 to force ectopic expression in developing photoreceptors. Resulting adults had rough eyes (data not shown), indicating problems with eye development. Staining for photoreceptor nuclei in eye discs or co-expression of a nuclear targeted GFP revealed severe disruption of nuclear positioning: many photoreceptor nuclei were found in basal sections, and distribution of nuclei in apical sections was irregular (Fig. 4B). Nuclear mislocalization was highly penetrant when using the GMR-Gal4 driver (42 out of 42 eye discs examined had disruptions similar to Fig. 4B). These defects are reminiscent of Klar α loss-of-function mutations. No nuclear mispositioning was observed in animals homozygous for *klar^SC1^* or animals carrying only the Gal4 drivers (data not shown).

Dramatic alteration of nuclear positioning was also observed when we drove ectopic expression in the female germ line, using the matα4-Gal4-VP16 driver (Fig. 4C). In the wild type, the oocyte nucleus is located posteriorly in stage 6 egg chambers and relocates to the dorsal anterior corner in stages 7 and 8 [29]. Proper nuclear positioning depends on the microtubule motors kinesin-1 and cytoplasmic dynein [30], but is not altered in any of the *klar* mutants examined to date [17], [22], including *klar^SC2^* (data not shown). Ectopic expression from the *EY13781* element resulted in drastically increased Klar levels, as judged by Klar-C immunostaining (Fig. 4C) and frequent displacement of the oocyte nuclei away from the anterior-dorsal corner (in about half of the stage 10 egg chambers: 12 out of 24 oocytes examined). Thus, ectopic expression of Klar isoforms from the P_δ/ε_ promoter can drastically affect nuclear position in multiple tissues.

## Discussion

Klar is broadly expressed during *Drosophila* development, and mutational analysis suggests that it is important for many distinct processes, from nuclear positioning in larval photoreceptors to size control of the apical membrane in cells of the embryonic salivary gland. A long-standing puzzle has been how this one regulator can impact so many specific processes.

It was previously suggested that isoform variation allows the organism to generate distinct versions of Klar with distinct functions. Indeed, alternative splicing generates Klar messages with one of two 3′ ends (exon 15ext versus exons 16,17,18). At the protein level, these alternative 3′ sequences result in C-termini that target the Klar proteins to distinct intracellular locations: the KASH domain (encoded in exon 18) targets the protein to the nuclear envelope; the LD domain (encoded in exon 15ext) targets the protein to lipid droplets [1], [9], [17], [18], [21].

In this manuscript, we demonstrate that further isoform complexity is achieved by variation of N-terminal sequences: two sets of promoters (P_α/β_, P_δ/ε_) appear to be active at different times and in different tissues, giving rise to forms of Klar that include (α, β) or lack (δ, ε) a ∼ 68 kDa N-terminal region. Although the function of this 68 kDa domain has not yet been deciphered, it presumably has specific interaction partners and imparts unique properties to Klar α and β. Since this variable N-terminal domain is part of the region of Klar proposed to interact with the microtubule motors kinesin-1 and dynein [9], [18], it will be interesting to determine if the presence of this domain modulates the binding or activity of one or both motors.

Currently, the biological function of the two new isoforms δ and ε is unknown. Robust expression in the embryonic and larval nervous system might suggest a neuron-related role. However, loss-of-function studies have not yet revealed any obvious defects in animals lacking these isoforms. It is possible that δ and ε act redundantly with other Klar isoforms or that defects are too subtle to be detected with our assays. For example, if these Klar isoforms were to modulate the speed or timing of nuclear migration in photoreceptors, it will require live imaging of these migration events to detect the phenotypes.

Nevertheless, our ectopic expression experiments show that Klar δ and/or ε can have potent activities when overabundant. In particular, they cause severe mislocalization of nuclei in larval photoreceptors and in oocytes. In larval photoreceptors, Klar δ/ε overexpression mimics Klar α loss of function, and thus these isoforms may interfere with the function of Klar α. In oocytes, loss of Klar α function does not affect nuclear position, so ectopic Klar δ/ε must target some other pathway. As the mechanism of nuclear positioning in oocytes remains poorly understood, ectopic expression of Klar δ/ε may provide a new tool to dissect it. An intriguing speculation is that ectopically expressed Klar δ recruits the machinery (such as microtubule motors) necessary for migration to the oocyte nucleus at a time when the oocyte usually remains stably anchored.

## Materials and Methods

### Fly stock

Oregon R was used as the wild-type stock. The mutant alleles *klar^mBP^, klar^mBX13^, klar^mBX14^* [16], *klar^1^* [2], *klar^YG3^* [9] and the deficiency *Df(3L*)*emc^E12^* have been described previously. The *klar^mBP^* allele is carried on a translocation chromosome that is homozygous lethal; thus, the full genotype of animals referred to as *klar^mBP^* in the text is *klar^mBP^*/*Df(3L)emc^E12^*. *EY13871*, a P-element insertion near the P_δ/ε_ promoter, was obtained from the Bloomington Stock Center and is referred to in the text as allele *klar^SC1^*. Imprecise excision of this P-element yielded *klar^SC2^*. For ectopic expression studies, Gal4 drivers matα4-Gal4-VP16 (female germ-line) and elav-Gal4 and GMR-Gal4 (eye imaginal discs) were employed. In some cases, we employed a chromosome that carries both GMR-Gal4 and UAS-NLS-GFP to mark the photoreceptor nuclei in living tissues.

### RT-PCR

Total RNA was isolated from embryos, larvae, pupae, and adult flies using Trizol reagent according to the manufacturer's instructions (Invitrogen). Larval instars were recognized by size: 1st instar <1 mm, 2nd between 1mm and 2mm; 3rd instar larva >5 mm). 2 µg RNA was used for primary cDNA synthesis with reverse transcriptase (Promega) at 37°C for 90 min in a total of 20 µl reaction volume. 1 µl primary cDNA synthesized was used for PCR reactions in a total volume of 50 µl. Exon specific primers as shown in Fig. 1A were used to detect specific Klar messages. Primer sequences available upon request.

### Western blotting

Embryos were collected, aged, and heat fixed as described [9]. For the Westerns in Fig. 2B and C, fixed embryos were visually sorted to obtain certain stages. Klar and Khc were detected by Western blotting as described previously [9], [18], [31].

### Immunostaining

Embryos, larval tissues, and ovaries were prepared and heat fixed as described [9]. Immunostaining with Klar-M (1∶50), Klar-C (1∶5), and anti-Elav (1∶500) was performed as described [9]. Images were acquired on Leica TCS SP2 or SP5 confocal microscopes.

## References

[1] Fischer JA, Acosta S, Kenny A, Cater C, Robinson C, et al (2004) Drosophila klarsicht has distinct subcellular localization domains for nuclear envelope and microtubule localization in the eye. Genetics 168: 1385–1393. 15579692 10.1534/genetics.104.028662 PMC1448802

[2] Welte MA, Gross SP, Postner M, Block SM, Wieschaus EF (1998) Developmental regulation of vesicle transport in Drosophila embryos: forces and kinetics. Cell 92: 547–557. 9491895 10.1016/s0092-8674(00)80947-2

[3] Starr DA, Fridolfsson HN (2010) Interactions between nuclei and the cytoskeleton are mediated by SUN-KASH nuclear-envelope bridges. Annu Rev Cell Dev Biol 26: 421–444. 20507227 10.1146/annurev-cellbio-100109-104037 PMC4053175

[4] Meyerzon M, Fridolfsson HN, Ly N, McNally FJ, Starr DA (2009) UNC-83 is a nuclear-specific cargo adaptor for kinesin-1-mediated nuclear migration. Development 136: 2725–2733. 19605495 10.1242/dev.038596 PMC2730402

[5] Roux KJ, Crisp ML, Liu Q, Kim D, Kozlov S, et al (2009) Nesprin 4 is an outer nuclear membrane protein that can induce kinesin-mediated cell polarization. Proc Natl Acad Sci U S A 106: 2194–2199. 19164528 10.1073/pnas.0808602106 PMC2650131

[6] Mosley-Bishop KL, Li Q, Patterson K, Fischer JA (1999) Molecular analysis of the *klarsicht* gene and its role in nuclear migration within the differentiating cells of the Drosophila eye. Curr Biol 9: 1211–1220. 10556085 10.1016/s0960-9822(99)80501-6

[7] Patterson K, Molofsky AB, Robinson C, Acosta S, Cater C, et al (2004) The functions of klarsicht and nuclear lamin in developmentally regulated nuclear migrations of photoreceptor cells in the Drosophila eye. Mol Biol Cell 15: 600–610. 14617811 10.1091/mbc.E03-06-0374 PMC329262

[8] Fridolfsson HN, Ly N, Meyerzon M, Starr DA (2010) UNC-83 coordinates kinesin-1 and dynein activities at the nuclear envelope during nuclear migration. Dev Biol 338: 237–250. 20005871 10.1016/j.ydbio.2009.12.004 PMC2826220

[9] Guo Y, Jangi S, Welte MA (2005) Organelle-specific Control of Intracellular Transport: Distinctly Targeted Isoforms of the Regulator Klar. Mol Biol Cell 16: 1406–1416. 15647372 10.1091/mbc.E04-10-0920 PMC551502

[10] Myat MM, Andrew DJ (2002) Epithelial tube morphology is determined by the polarized growth and delivery of apical membrane. Cell 111: 879–891. 12526813 10.1016/s0092-8674(02)01140-6

[11] Elhanany-Tamir H, Yu YV, Shnayder M, Jain A, Welte M, et al (2012) Organelle positioning in muscles requires cooperation between two KASH proteins and microtubules. J Cell Biol 198: 833–846. 22927463 10.1083/jcb.201204102 PMC3432764

[12] Dworkin I, Kennerly E, Tack D, Hutchinson J, Brown J, et al (2009) Genomic consequences of background effects on scalloped mutant expressivity in the wing of Drosophila melanogaster. Genetics 181: 1065–1076. 19064709 10.1534/genetics.108.096453 PMC2651043

[13] Zhao T, Gu T, Rice HC, McAdams KL, Roark KM, et al (2008) A Drosophila gain-of-function screen for candidate genes involved in steroid-dependent neuroendocrine cell remodeling. Genetics 178: 883–901. 18245346 10.1534/genetics.107.082487 PMC2248363

[14] Myat MM, Lightfoot H, Wang P, Andrew DJ (2005) A molecular link between FGF and Dpp signaling in branch-specific migration of the Drosophila trachea. Dev Biol 281: 38–52. 15848387 10.1016/j.ydbio.2005.02.005 PMC2827869

[15] Harbison ST, Chang S, Kamdar KP, Mackay TF (2005) Quantitative genomics of starvation stress resistance in Drosophila. Genome Biol 6: R36. 15833123 10.1186/gb-2005-6-4-r36 PMC1088964

[16] Fischer-Vize JA, Mosley KL (1994) Marbles mutants: uncoupling cell determination and nuclear migration in the developing Drosophila eye. Development 120: 2609–2618. 7956836 10.1242/dev.120.9.2609

[17] Xie X, Fischer JA (2008) On the roles of the Drosophila KASH domain proteins Msp-300 and Klarsicht. Fly (Austin) 2: 74–81. 18820482 10.4161/fly.6108

[18] Yu YV, Li Z, Rizzo NP, Einstein J, Welte MA (2011) Targeting the motor regulator Klar to lipid droplets. BMC Cell Biol 12: 9. 21349165 10.1186/1471-2121-12-9 PMC3051913

[19] Zhang Q, Skepper JN, Yang F, Davies JD, Hegyi L, et al (2001) Nesprins: a novel family of spectrin-repeat-containing proteins that localize to the nuclear membrane in multiple tissues. J Cell Sci 114: 4485–4498. 11792814 10.1242/jcs.114.24.4485

[20] Starr DA, Fischer JA (2005) KASH 'n Karry: the KASH domain family of cargo-specific cytoskeletal adaptor proteins. Bioessays 27: 1136–1146. 16237665 10.1002/bies.20312

[21] Kracklauer MP, Banks SM, Xie X, Wu Y, Fischer JA (2007) Drosophila klaroid encodes a SUN domain protein required for Klarsicht localization to the nuclear envelope and nuclear migration in the eye. Fly (Austin) 1: 75–85. 18820457 10.4161/fly.4254

[22] Technau M, Roth S (2008) The Drosophila KASH domain proteins Msp-300 and Klarsicht and the SUN domain protein Klaroid have no essential function during oogenesis. Fly (Austin) 2: 82–91. 18820478 10.4161/fly.6288

[23] Arbeitman MN, Furlong EE, Imam F, Johnson E, Null BH, et al (2002) Gene expression during the life cycle of Drosophila melanogaster. Science 297: 2270–2275. 12351791 10.1126/science.1072152

[24] Ohler U, Liao GC, Niemann H, Rubin GM (2002) Computational analysis of core promoters in the Drosophila genome. Genome Biol 3: RESEARCH0087. 12537576 10.1186/gb-2002-3-12-research0087 PMC151189

[25] Ohler U (2006) Identification of core promoter modules in Drosophila and their application in accurate transcription start site prediction. Nucleic Acids Res 34: 5943–5950. 17068082 10.1093/nar/gkl608 PMC1635271

[26] Roiha H, Rubin GM, O'Hare K (1988) P element insertions and rearrangements at the singed locus of Drosophila melanogaster. Genetics 119: 75–83. 2840331 10.1093/genetics/119.1.75 PMC1203347

[27] McQuilton P, St Pierre SE, Thurmond J (2011) FlyBase 101 – the basics of navigating FlyBase. Nucleic Acids Res 40: D706–D714. 22127867 10.1093/nar/gkr1030 PMC3245098

[28] Wolff T, Ready DF (1993) Pattern formation in the Drosophila retina. In: Bate M, Martinez-Arias A, editors. The Development of Drosophila melanogaster. Cold Spring Harbor, NY: Cold Spring Harbor Laboratory Press. 1277–1326.

[29] van Eeden F, St Johnston D (1999) The polarisation of the anterior-posterior and dorsal-ventral axes during Drosophila oogenesis. Curr Opin Genet Dev 9: 396–404. 10449356 10.1016/S0959-437X(99)80060-4

[30] Duncan JE, Warrior R (2002) The cytoplasmic Dynein and Kinesin motors have interdependent roles in patterning the Drosophila oocyte. Curr Biol 12: 1982–1991. 12477386 10.1016/s0960-9822(02)01303-9

[31] Shubeita GT, Tran SL, Xu J, Vershinin M, Cermelli S, et al (2008) Consequences of motor copy number on the intracellular transport of kinesin-1-driven lipid droplets. Cell 135: 1098–1107. 19070579 10.1016/j.cell.2008.10.021 PMC2768369

